# The Mechanism of Enhanced Insulin Amyloid Fibril Formation by NaCl Is Better Explained by a Conformational Change Model

**DOI:** 10.1371/journal.pone.0027906

**Published:** 2011-11-21

**Authors:** Mahvish Muzaffar, Atta Ahmad

**Affiliations:** Department of Biological Chemistry and Life Sciences Institute, University of Michigan, Ann Arbor, Michigan, United States of America; Russian Academy of Sciences, Institute for Biological Instrumentation, Russian Federation

## Abstract

The high propensity of insulin to fibrillate causes severe biomedical and biotechnological complications. Insulin fibrillation studies attain significant importance considering the prevalence of diabetes and the requirement of functional insulin in each dose. Although studied since the early years of the 20^th^ century, elucidation of the mechanism of insulin fibrillation has not been understood completely. We have previously, through several studies, shown that insulin hexamer dissociates into monomer that undergoes partial unfolding before converting into mature fibrils. In this study we have established that NaCl enhances insulin fibrillation mainly due to subtle structural changes and is not a mere salt effect. We have carried out studies both in the presence and absence of urea and Gdn.HCl and compared the relationship between conformation of insulin induced by urea and Gdn.HCl with respect to NaCl at both pH 7.4 (hexamer) and pH 2 (monomer). Fibril formation was followed with a Thioflavin T assay and structural changes were monitored by circular dichroism and size-exclusion chromatography. The results show salt-insulin interactions are difficult to classify as commonly accepted Debye-Hückel or Hofmeister series interactions but instead a strong correlation between the association states and conformational states of insulin and their propensity to fibrillate is evident.

## Introduction

Protein aggregation has serious biochemical, physiological and clinical implications [1], [2]. More than 20 diseases have been found to owe their etiology to amyloid aggregation of proteins [2]. Amyloid protein deposits consist of linear fibrils ∼20 Å wide and 1–6 nm long [3] that possess cross β structure [4], [5] and exhibit birefringence of bound Congo red under polarized light [6]. The observation that stable globular proteins form fibrils on destabilization [7] and the ability of proteins or polypeptides to form fibrils independent of their origin, class or function [8], [9], [10], [11] have led to a common belief that conformational switches from the native state are responsible for fibrillation [12], [13]. This is supported by reports in which the propensity of a protein to fibrillate is enhanced under destabilizing conditions owing to buildup of partially-folded intermediates, such as, by denaturants [14], mutations [15], [16], co-solvents [17], temperature [18], pH [19] or other [20], [21], [22].

Insulin is a 51-residue hormone that exists as equilibrium mixture of hexamer, tetramer, dimer and monomer in solution. The protein is predominantly hexameric in the presence of zinc at neutral pH (also the biological storage form), monomeric in 20% acetic acid (pH 1.8) and dimeric in 20 mM HCl (pH 2.0) [14]. Insulin forms amyloid-like fibrils [23], [24] that pose a variety of problems in its biomedical and biotechnological applications (especially in insulin pumps). Amyloid deposits of insulin have been observed both in patients with type II diabetes and in normal aging, as well as after subcutaneous insulin infusion and after repeated injection. Recent literature indicates increasing incidence of insulin amyloid in clinical situations [25], [26], [27], [28], [29], [30], [31]. Injected insulin seems to form fibrils irrespective of the site of injection. For example, amyloid insulin was observed in thighs [26], [27], shoulders [28], arms [30], and abdominal walls [29], [30], [31] of patients at or around the site of repeated injections. Amyloid in these cases was also observed to be formed irrespective of the type [25], [26] or source [30] of insulin. The studies of insulin aggregation therefore have immense application in improving insulin therapy prescribed to diabetic patients. This is important, as the number of people suffering from this disease is immense. In 2000, 171 million people were estimated to be suffering from diabetes and the number is expected to increase to 300 million in 2030 [32]. It is important to mention here that the clinical cases of insulin amyloid may seem few as detected in the developed countries with advancement in pumps, pens and insulin preparations but at the same time it is rational to think how these incidences would translate in the underdeveloped or developing world, especially countries loaded with the population burden.

Studies on insulin aggregation started in the early years of 20^th^ century. Soon after pure insulin was purified by Abel [33], insulin was observed to form precipitates at high temperature and low pH [34], [35]. Waugh showed that the precipitates were fibrilar in nature with EM and birefringence experiments [36] and that the process of fibrillation could be carried out at room temperature with seeding [23]. A number of investigations have been undertaken to elucidate the mechanism of insulin fibrillation [24], [37], [38], [39]. It has been proposed that insulin fibrillation occurs through dissociation of oligomers into monomers and that the monomer undergoes a structural change to a conformation having strong propensity to fibrillate [23], [24], [26], [40], [41]. In our previous studies we have discussed in detail the involvement of conformational modifications of insulin towards its fibrillation[14], [20], [42], [43], [44]. Insulin fibrillation was accelerated by low to moderate concentrations of guanidine hydrochloride (Gdn.HCl) at physiological pH due to hexamer dissociation [14]. The predominant species under these conditions was a partially folded monomer that was present even at >6 M Gdn.HCl. Similarly for monomeric insulin (pH 2), Gdn.HCl induced partially folded conformations enhanced insulin fibrillation [14]. When treated with urea, the rate of insulin fibrillation increased with increase in the accumulation of modified monomeric form of protein that sustained even above 8.0 M urea. Surprisingly, addition of low concentrations of urea at low pH resulted in generation of a compacted conformational state of insulin that exhibited resistance towards fibrillation [42]. Dissecting further we found that both chain-A and chain-B of insulin were capable of forming fibrils individually [44]. Thus, a minimum pathway for insulin fibrillation was postulated i.e., hexamer→monomer→partially unfolded monomer→fibrils [14], [42].

In the present study, we have tried to investigate the effect of NaCl on the fibrillation of insulin and to propose that structural changes caused by NaCl are the real contributors to enhanced fibrillation observed in the presence of NaCl. We carried out studies with or without urea and Gdn.HCl and have attempted to compare the relationship between conformation of insulin induced by NaCl with respect to urea and Gdn.HCl and their propensity to fibrillate, both at pH 7.4 (hexameric) and pH 2 (monomeric). Fibrillation kinetics was followed with a Thioflavin T (ThT) binding fluorescence assay [45] and circular dichroism and size-exclusion chromatography was used to monitor the structural changes taking place during the course of study. Taken together, the data shows a strong correlation between the association and conformational states of insulin and their propensity to fibrillate. The results, in conjunction with earlier studies on insulin fibrillation [14], [23], [43], [44], enhance our understanding of insulin fibrillation and will be useful in formulating insulin therapies.

## Materials and Methods

### Preparation of samples

Stock solutions of human insulin (2–10 mg/mL) were prepared fresh before use in 25 mM HCl pH 1.6. For experiments starting with hexameric insulin, aliquots of 1 M HEPES buffer were added to obtain a final concentration of 50 mM HEPES and adjusted to pH 7.4 using NaOH. For studies of monomeric insulin, a stock solution was freshly prepared by dissolving human insulin in 20% acetic acid (pH 2.0). The concentration of insulin was determined by using an extinction coefficient of 1.0 for 1 mg/mL at 276 nm [46]. The concentration of urea and Gdn.HCl solutions was obtained by refractive index measurement. Stock (1 mM) solutions of ThT were prepared by dissolving ThT in doubly distilled water and the concentration determined using a molar extinction coefficient of 24,420 M^−1^ cm^−1^ at 420 nm. ThT was stored at 4°C, protected from light. Aliquots from the insulin stock solutions were added to the solutions containing the desired concentrations of urea, Gdn.HCl and NaCl either individually or in combination to in a way to reach final concentrations of 2 mg/mL protein, 50 mM HEPES and 20 µM ThT for studying the effect of various solutions on conformation and fibrillation. In the case of studies at pH 2, aliquots were added to each sample to reach a final concentration of 20% acetic acid, 2 mg/mL human insulin, 20 µM ThT and varying urea, Gdn.HCl or NaCl concentrations.

### Kinetics of fibrillation

ThT fluorescence was monitored for each sample in a 96-well plate in a Fluoroskan Ascent CF fluorescence plate reader (Lab Systems) at 37°C or 40°C and shaking at 760 rpm. The plate was sealed with Mylar plate sealing film (Thermo Labsystems, MA, USA). The excitation wavelength was 444 nm and the emission measured at 485 nm. Plates were continuously shaken for the entire period of study at a rotation diameter of 1 mm and the integration period was fixed at 20 ms for each well. Five replicates, corresponding to five wells for each sample, were used to minimize well-to-well variation. Results from >4 similar profiles were averaged for the final results at each urea concentration. The kinetic profiles were analyzed for lag time by curve fitting, using SigmaPlot software [14], [21].

### Circular Dichroism (CD)

CD spectra were taken at 25°C on an AVIV 60DS circular dichroism spectrophotometer (Aviv Associates, Lakewood, NJ). Varying concentrations of urea, NaCl and Gdn.HCl, individually or in combination, were prepared in 50 mM HEPES pH 7.4 or 20% acetic acid, respectively. Aliquots from insulin stock solutions were mixed to obtain a final concentration of 2 mg/mL protein. To eliminate contributions from buffer, solutions without protein were prepared in the same manner as above and their spectra subtracted from the protein spectra. Samples were incubated for two hours before collecting the spectra. Near-UV spectra were recorded using a step size of 0.5 nm and a bandwidth of 1.5 nm in a cell of 0.5 cm path length. Phase diagrams were constructed by plotting molar ellipticity at 273 nm against 252 nm as reported in our previous studies [14].

### Size exclusion chromatography (SEC)

SEC was performed using a Superose 12 HR 10/30 column from Pharmacia Biotech. (Exclusion limit 2×106 Mr). The column was run on an FPLC GP 250-P500 (Pharmacia Biotech) instrument at a flow rate of 0.5 mL/min with detection at 280 nm. Human insulin, 2 mg/mL was incubated in 50 mM HEPES pH 7.4, with and without urea or a combination of urea+NaCl, urea+Gdn.HCl for 2 hours at 25°C. Aliquots of 200 µL of each sample were loaded on the column, which was equilibrated with 50 mM HEPES buffer containing the desired concentration of urea, urea+NaCl or urea+Gdn.HCl prior to the loading of sample. Aprotonin (6.5 kD) and cytochrome c (12.3 kD), corresponding to monomeric and dimeric insulin, were run as markers in the presence of 0.5 M urea, 50 mM HEPES pH 7.4. Since no significant changes in these proteins have been observed in 0.5 M urea, these markers were assumed to be globular under these conditions. The observed elution volume of 15.0 mL and 14.0 mL (data not shown) for aprotonin and cytochrome c is consistent with their size.

## Results

### ThT studies

**Figure 1:**
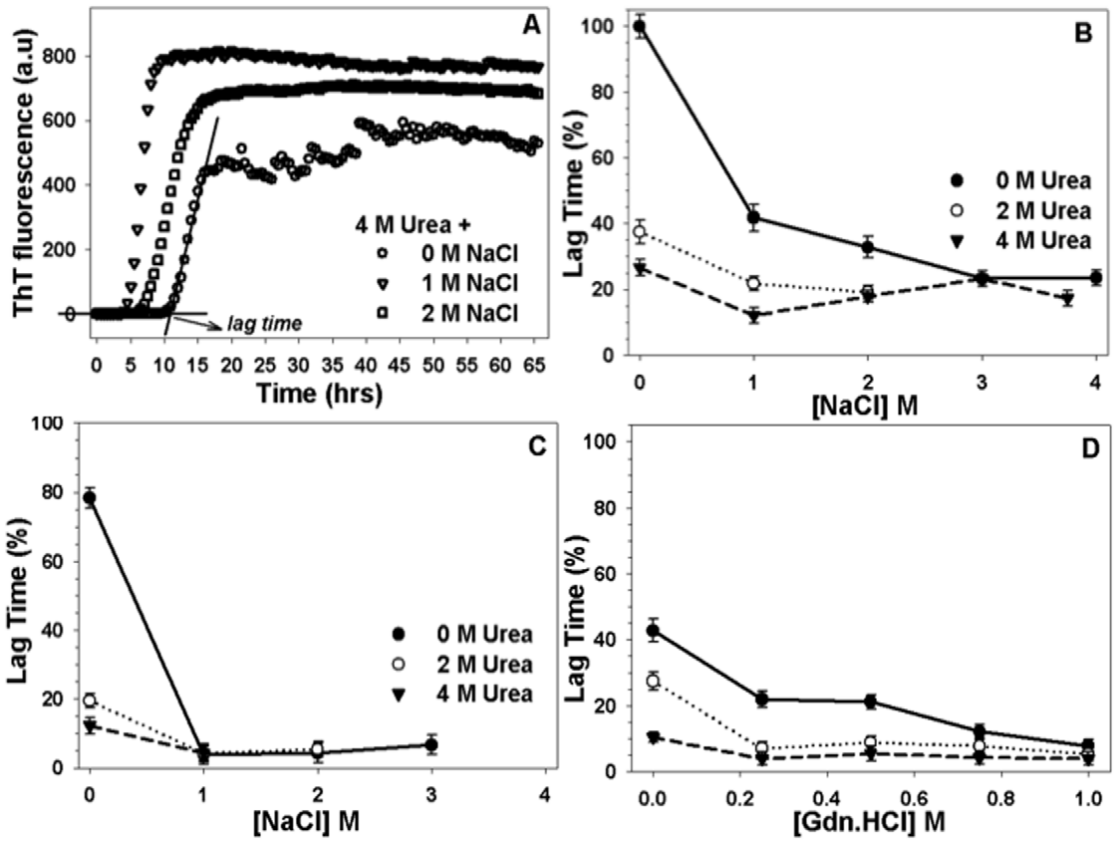
Kinetics of insulin fibrillation. Panel A shows representative sigmoidal curves of insulin fibrillation obtained at increasing NaCl concentrations in the presence of 4 M urea pH 7.4. The profiles are a plot of ThT emission max at 482 nm with respect to time. Panel B shows changes in the lag time of insulin fibrillation obtained from ThT plots at increasing NaCl concentrations in the presence of either 0 M (circles), 2 M (inverted triangles) or 4 M urea (squares) at pH 7.4. Panel C exhibits changes in the lag time with increasing NaCl concentrations in the presence of either 0 M (circles), 2 M (inverted triangles) or 4 M urea (squares) at pH 2. Panel D shows changes in the lag time with increasing Gdn.HCl concentrations in the presence of either 0 M (circles), 2 M (inverted triangles) or 7 M urea (squares) at pH 7.4.

We monitored fibrillation of insulin with ThT fluorescence as we have previously used this assay to monitor fibrillation of different proteins [14], [21], [22]. Fig. 1A shows the representative profiles of ThT fluorescence obtained after incubating insulin (2 mg/mL) in the presence of constant 4 M urea and varying concentrations of NaCl. The sigmoid curves exhibit characteristic ‘lag phase’, ‘elongation phase’, and ‘maturation phase’ of fibrillation kinetics. Changes in the lag phase have been shown to directly reflect the kinetics of fibrillation [21] compared to elongation and maturation phase [42]. A decrease in the lag time indicates faster fibrillation [14], [21], [42]. Fig. 1B summarizes changes in the lag phase of insulin (calculated according to [21]) in the presence of different NaCl concentrations with or without urea at pH 7.4. In the absence of urea, lag time of hexameric insulin decreases by more than 50% upon addition of 1 M NaCl and decreased further with increasing NaCl concentrations. Lag time reduced to ∼25% of the control in the presence of 4 M NaCl. In the presence of 2 M urea, lag time was observed to decrease by 60% in agreement with the previously published results [42]. Lag time was observed to further reduce upon addition of increasing concentrations of NaCl at constant 2 M urea. As expected, a decrease in the lag time by more than 70% was observed for 4 M urea treated insulin, which further decreased upon addition of 1 M NaCl at constant 4 M urea. However, the lag time exhibited a slight increase upon further addition of NaCl (2 or 3 M NaCl) under these conditions. Taken together these results infer that the presence of NaCl further accelerates the rapid fibrillation induced by urea for insulin except for conditions with 4 M urea:2–3 M NaCl. Studies carried out in 20% acetic acid (pH 2, monomeric insulin) show a similar accelerated fibrillation taking place in the presence of NaCl and urea. For monomeric insulin we observed a 20% reduction in the lag time that reduced drastically by 80% after addition of 1 M NaCl. The limitations in ThT assay (of monitoring a kinetic association process) under these experimental conditions obscured exploring the differences in lag time on further addition of NaCl, however, the rate of fibrillation remained enhanced in the presence of all NaCl concentrations studied. In the absence of NaCl, 2 or 4 M urea increased fibrillation of insulin that increased further upon addition of NaCl. Overall, the presence of NaCl was observed to induce more intense fibrillation for monomeric insulin than for hexameric insulin. We also studied the effect of addition of Gdn.HCl to compare the effect of addition of NaCl (see also Figure S1). We have previously shown that addition of Gdn.HCl and urea results in the faster fibrillation for hexameric insulin [14], [42]. 2, 4 or 7 M urea exhibited enhanced fibrillation by itself. Addition of 0.25–1 M Gdn.HCl in the presence of 2 M urea induced faster fibrillation in insulin compared to urea alone. The fibrillating capacity further increased at higher urea concentrations in combination with 0.25–1 M Gdn.HCl.

### Near UV CD

**Figure 2:**
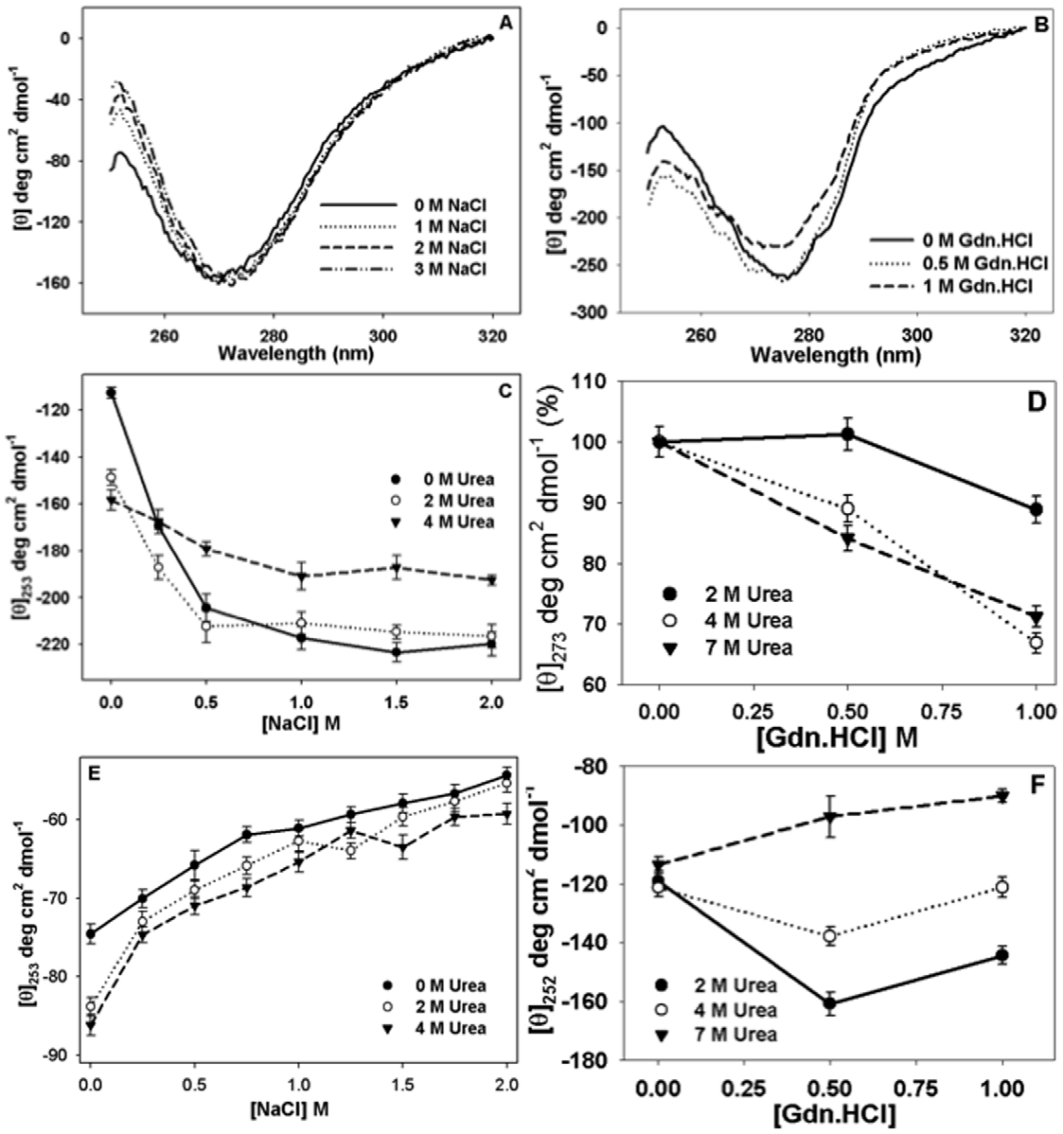
Structural changes of insulin. Panel A exhibits representative near-UV CD spectra of insulin at different NaCl concentrations at pH 2 and Panel B shows spectra at different Gdn.HCl concentrations at pH 7.4. Panel C shows changes in molar ellipticity at 252 nm with increasing NaCl concentration in the presence of either 0 M (filled circles), 2 M (open circles) or 4 M urea (inverted triangles) at pH 7.4. Panel D shows % changes in molar ellipticity at 273 nm with increasing Gdn.HCl concentration in the presence of either 2 M (filled circles), 4 M (open circles) or 7 M urea (inverted triangles) at pH 7.4 (For raw data of Panel D please refer to Figure S3). Panel E shows changes in molar ellipticity at 252 nm with increasing NaCl concentration in the presence of either 0 M (filled circles), 2 M (open circles) or 4 M urea (inverted triangles) at pH 2. Panel F shows changes in molar ellipticity at 252 nm with increasing Gdn.HCl concentration in the presence of either 2 M (filled circles), 4 M (open circles) or 7 M urea (inverted triangles) at pH 7.4.

The representative spectra of insulin in the presence of NaCl only and in a combination of urea:Gdn.HCl are shown in figure 2A & 2B, respectively. A characteristic negative peak at 276 nm (due to aromatic tyrosines) is observed for native insulin in all cases as reported earlier [14], [42]. For monomeric insulin, only the intensity of this peak was observed to be reduced. Figure 2A shows upon addition of NaCl subtle changes occurred in the peak at 276 nm, however, the peak around 252 nm underwent significant changes. Wavelength 252 nm has been attributed to disulfide bonds in CD spectra [47], [48]. Insulin possesses three disulfides. A plot of changes in intensity at 253 nm against the concentration of NaCl, pH 7.4, (Figure 2C) shows that the intensity of this peak decreases upon addition of increasing NaCl concentrations indicating a structural rigidity induced around the disulfide bonds. Addition of urea (2 or 4 M) led to loss of structure around tyrosines (276 nm) in insulin but the disulfide environments were found to be more ordered matching our previous studies [42]. Presence of NaCl in addition to urea led to further orientation of the disulfides towards rigidity with the effect being more pronounced at 2 M urea than 4 M urea in combination with NaCl. The disulfide region of monomeric insulin (Figure 2E) exhibited the reverse trends. The intensity of peak at 253 nm exhibited an increase on addition of NaCl showing that the structure was getting more flexible up on addition of NaCl. Addition of urea (2 or 4 M) by itself led to ordering in the disulfide environments but in the presence of NaCl the significant dis-organization of structure around disulfides was observed. We thus can conclude while at pH 7.4 disulfide environments get rigid, at pH 2 a considerable flexibility in structure was attained upon addition of NaCl. We wanted to compare the effect of the urea:NaCl pair to the effects caused by Gdn.HCl or urea:Gdn.HCl. Since Gdn.HCl is a strong reagent we used only up to 1 M solutions. Additionally, this helped us overcome solubility problems and carry out studies in combination with 7 M urea. The sequence of addition of chaotropes did not seem to influence the end result (Figure S2). Figure 2B shows considerable changes in the shape and intensity of insulin spectra both around 252 and 276 nm at pH 7.4 with addition of Gdn.HCl (0.5 or 1 M). This indicates that unlike the localized effect by NaCl, Gdn.HCl induces global structural changes in insulin. To get a better insight we plotted the changes in the intensity at 273 nm as % changes against Gdn.HCl (for raw data please refer to Figure S3). The combination of denaturants resulted in 10% loss of structure compared to control insulin at 2 M urea:1 M Gdn.HCl. A 30% disruption was observed at 4 or 7 M urea:1 M Gdn.HCl. Interestingly, the changes in disulfide environment were not monotonous. In the presence of 2 or 4 M urea:0.5 M Gdn.HCl the loosening of structure around disulfide group was observed while addition of 1 M Gdn.HCl seemed to re-orient the disulfide towards the environment in control insulin. Contrary to this, in 7 M urea, addition of Gdn.HCl lead to increasing organization of structure around disulfides with the increase in Gdn.HCl concentration. In conclusion, all these experiments show subtle and gross structural changes taking place in insulin. At this stage, it can be arbitrarily assumed that insulin is in different conformational states depending on the concentration of each added chemical.

**Figure 3:**
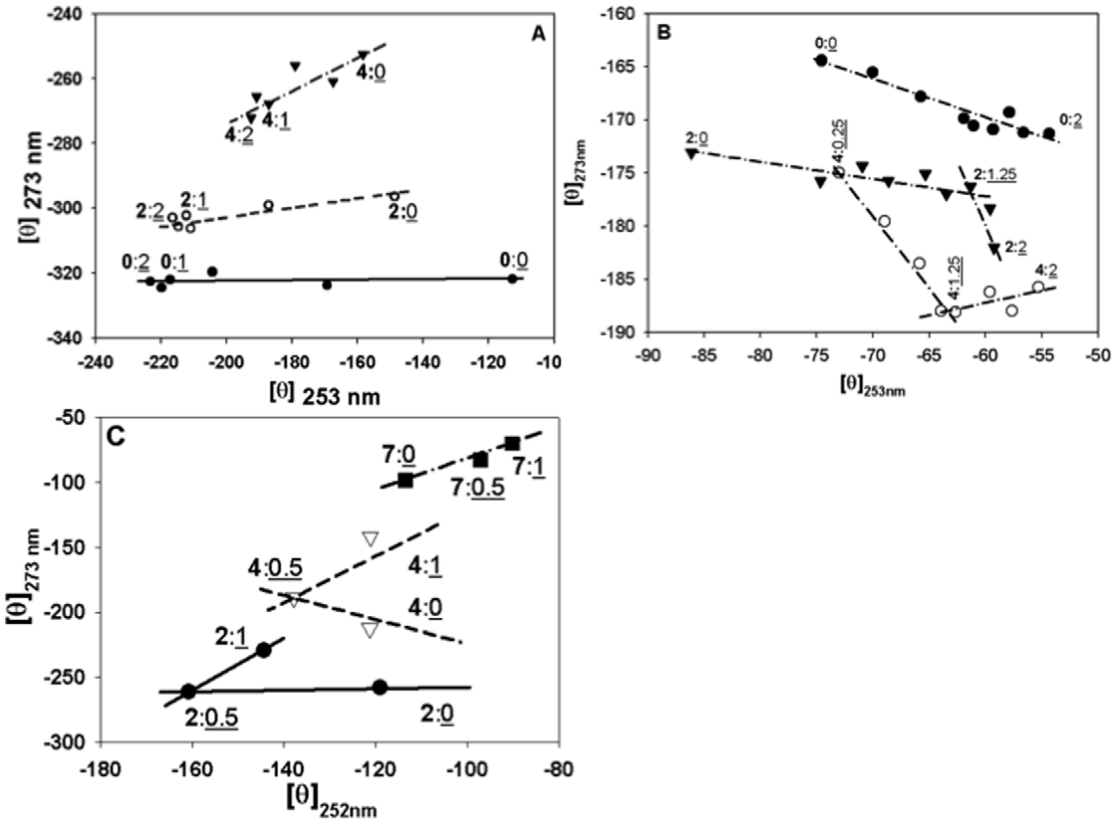
Phase diagram indicating the transitions taking place in the insulin. Diagrams have been obtained by plotting molar ellipticity at 252 and 273 nm from the near-UV CD. Numbers in panel A and B indicate molar concentration as urea (bold):NaCl (underlined) of the nearest point and in panel C that as urea(bold):Gdn.HCl (underlined) of the nearest point, respectively. Panel A exhibits a phase diagram for data obtained at pH 7.4 at different concentrations of urea and NaCl. Panel B exhibits a phase diagram obtained at pH 2 in the presence of different concentrations of urea and NaCl. Panel C exhibits a phase diagram for data obtained at pH 7.4 at different concentrations of urea and Gdn.HCl.

To represent the structural perturbations observed in full near UV CD spectrum better, we plotted the phase diagrams for CD data. Phase diagrams have been used to simplify complex data obtained in fluorescence and CD experiments [14], [42], [49], [50]. Each linear segment represents all-or-none transition between two conformations. Phase diagrams with present data were obtained by plotting molar ellipticity at 273 nm vs 253 nm. These were chosen, as these are the wavelengths were the changes occur or are supposed to occur. In each graph of Figure 3 the concentration of urea:NaCl or urea;Gdn.HCl is shown next to the data point. The concentration of urea has been shown in bold and that of NaCl or Gdn.HCl has been shown underlined in the figure. Figure 3A shows a single transition taking place in all the three sets of studies indicating the existence of at least two structurally different states in each set (6 states in all). Monomeric insulin (Figure 3B) shows structural transformation between two states in the presence of NaCl only but in combination with urea NaCl results in formation of an additional conformation at 1.25 M (8 states in this set). Gdn.HCl and urea by itself have been shown to induce multiple conformational states in insulin [14], [42]. In Figure 3C presence of Gdn.HCl in combination with 2 or 4 M urea results in the formation of three structurally different states in each set. Interestingly, in the mostly unfolded insulin at 7 M urea, addition of Gdn.HCl is able to unfold it into another distinct conformation. Taken together the CD data analysis emphasizes that NaCl by itself is able to induce structural changes in insulin, especially around the disulfide region and in combination with urea is able to further modify its structure.

### SEC studies

**Figure 4:**
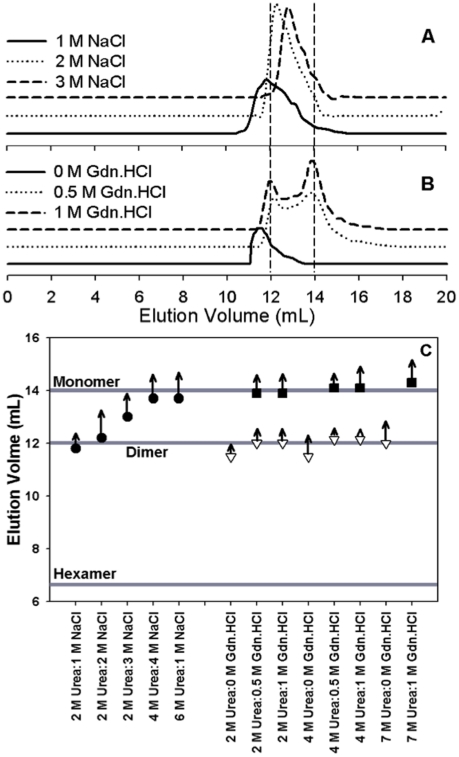
SEC studies of insulin. Panel A shows representative SEC profiles (absorbance 280 nm) of insulin obtained in the presence of varying concentrations of NaCl and urea at pH 7.4, (solid line) 2 M urea:1 M NaCl, (dotted line) 2 M urea:2 M NaCl, (dashed line) 2 M urea:3 M NaCl. Panel B shows representative SEC profiles obtained in the presence of varying concentrations of Gdn.HCl and Urea at pH 7.4, (solid line) 2 M urea:0 M Gdn.HCl, (dotted line) 2 M urea:0.5 M Gdn.HCl, (dashed line) 2 M urea:1 M Gdn.HCl. Vertical dotted lines on Panel A and B corresponding to elution volumes at max peak heights. Panel C provides overall SEC analysis. The three solid lines act as markers for the monomeric, dimeric and hexameric insulin based on SEC elution volumes (mL). Symbols show the elution volume of the associated species of insulin (mono- or multi-meric state) and the arrows on the symbols show the intensity (scaled down in millimeters) of the corresponding peaks in the range of this study. Filled circles represent elution volumes (mL) at different urea and NaCl concentrations as shown along the ordinate axis corresponding to each data point. Filled squares and inverted triangles represent the elution volumes (mL) of insulin treated with different concentrations of urea and Gdn.HCl shown accordingly on the ordinate axis.

Another way to compare the structural changes induced by NaCl, NaCl:urea or urea:Gdn.HCl is by SEC. Figure 4A shows the representative chromatograms of insulin. Vertical dotted lines at 12 mL and 14 mL correspond to the elution volumes of cytochrome c (12.3 kD, ∼insulin dimer) and aprotonin (6 kD, ∼insulin monomer), respectively. We have extensively analyzed and characterized similar SEC studies for insulin previously [14], [42]. The elution volume of insulin at pH 7.4 (hexamer) was 6.4 mL (not shown). In the presence of 2 M urea insulin eluted with maximum peak intensity at 11.5 mL indicating a form larger than dimer. Addition of 0.5 M Gdn.HCl led to the dissociation of this structure to a dimeric and monomeric form as evidenced by the change in their elution volumes corresponding to standards for dimeric and monomeric insulin. Increasing Gdn.HCl concentration to 1 M led to shift in the dissociation equilibrium towards the monomeric form of insulin. Figure 4C summarizes the SEC results obtained at other combinations of Gdn.HCl and urea. The symbols in the figure indicate elution volumes and the arrows on top of symbols indicate the intensity of the peak corresponding to that elution volume. The length of each arrow (scaled down in millimeters) corresponds to the max height of the particularly peak at that elution volume (does not imply quantitative analysis). For example, for 2 M urea:0 M Gdn.HCl a peak corresponding to species larger than dimer form was observed. Addition of Gdn.HCl (0.5 or 1 M) dissociated insulin into a dimer and monomer with slight changes in intensities. Compared to this at 4 M urea the presence of 1 M Gdn.HCl caused pronounced changes in the intensity of monomer compared to another point in this set. Addition of 1 M Gdn.HCl with 7 M urea shifted the equilibrium absolutely to monomeric insulin. However, the studies carried out in the presence of NaCl were not so clear. Addition of 1 M NaCl in combination of 2 M urea (Figure 4A) increased the elution volume slightly but the size of the peak increased indicating either a loosening of a structure or dissociation into rapidly exchanging states. Increasing NaCl concentration to even 3 M did not yield a clear dissociation of insulin but instead broad peaks eluting between the standard proteins were observed. Based on our earlier studies [14], [42] we assume a scenario where a fast equilibrium between tetramer and dimer is taking place in the former case and between a monomer and dimer in the latter two cases, respectively. The same effect was observed at other combinations of urea and NaCl (Figure 4C). Even at 4 M urea and 4 M NaCl or 6 M urea and 1 M NaCl no distinct dissociation into monomer or dimer was observed.

## Discussion

The concept of ‘conformational disease’ holds its etiology to the existence of the processes of misfolding or toxic folding. *In vitro* studies on suspect proteins have mostly shown faster propensities to fibrillate in altered conformations with respect to their native state including the class of natively unfolded proteins [51]. In the case of unfolded proteins it seems straight forward that addition of compounds that interact (directly or indirectly) with the backbone or shield (repulsive/attractive) moieties result in significant effect on the association of the molecule concerned. But with globular proteins unless the added compound/condition possesses strong disruptive properties a significant change in its association probability are hard to fathom.

The studies become more interesting as the dichotomy of solvents get increasingly blurred. For example, Gdn.HCl and urea, known chaotropes, modify the structure of insulin. These modified forms have been found to be aggregation-prone intermediate species that stimulate the fibrillation kinetics of insulin [14], [42]. However, the effect of addition of NaCl or other salts on insulin cannot be explained as clearly. Salts in general have been observed to mediate through ion interactions - Debye-Hückel screening [52] or electroselectivity series [53], [54], or by influencing water-solute interactions - Hofmeister series [55], [56], [57]. Different proteins have been shown to interact with salts in their own unique way during fibrillation process. β-microglobulin [58] and glucagon [59] have been observed to follow electroselectivity series of interactions while α-Synuclein has been observed to follow Hofmeister series of interactions [60] with salts. The identification of real interactions becomes more complex as Aβ has been reported to follow both Hofmeister and anion binding series [61]. In addition, yeast PrP Sup35 followed Hofmeister series [62] while the mouse PrP has been observed to follow the electroselectivity series [63]. Further, it has been reported that same salt shows a biphasic effect, that is, at low concentrations it induce opposite effect of that induced at higher concentrations under similar conditions [58], [64]. Thus, as acknowledged by Munishkina et al., [60], the salt effects are a combination of the electrostatic and solvent interactions besides other exogenous and endogenous factors. We believe the one additional effect of salts that has been overlooked so far is their contribution to the structural modification of the protein and this may be a bigger player in rendering the susceptibility of protein towards fibrillation. This statement is supported by finding where glucose oxidase has been found to undergo structural changes upon addition of monovalent salts leading to the stability of this enzyme [65]. We will try to explain our results in light of these arguments as below. We have used high amounts of salt mainly to get an overall picture for our studies. Although not insulin specific, the high concentration salt studies should not be considered physiologically irrelevant as during anti-diuresis the medullar fluid similarities can increase up to ∼2.0 M NaCl (equivalent of 3800 mOsmol l^−1^) in mammalian kidneys (reviewed in [66]).

It has been proposed that the Debye-Hückel screening effect should be influenced by the ionic strength whereas the Hofmeister series undergoes an inversion as a function of pH [60]. Also, NaCl has been shown to induce maximum fibrillating effect at a concentration of 200 mM whereas other sodium salts are able to reach similar changes at 4–60 times less concentration [58], [64]. Our ThT data at pH 2.0 with NaCl shows an enhancement in the fibrillation with the increase in the concentration of NaCl from lower concentrations (0.5 M) to higher (3 M). The trend continued to follow even in the presence of urea. On the other hand, Gdn.HCl at pH 7.4 exhibits a relatively gradual increase in ThT kinetics upon addition of Gdn.HCl both in the presence and absence of urea probably due to the narrow range of concentration included in this study (0–1 M). Taken alone this behavior then follows the Debye-Hückel rules of more screening with more ionic strength. However, at pH 7.4 insulin without urea compares well with the monotonous trend observed in the above-mentioned cases but in the presence of urea there are subtle deviations in that the effects on ThT kinetic seem to reverse above 2 M NaCl concentrations. This could be explained in terms of the structural changes induced by the salt much like the A states reported by Goto et al., [67]. Conversely, the ion reverses its effect upon change in the global structure of protein.

The changes observed in CD spectra are reproducible in NaCl, urea or Gdn.HCl thereby making the data robust. Also, if we compare it with our previously published CD profiles for insulin [14], [42] the changes in the data around 253 nm are significantly reproducable and exhibit a definite trend. The attempts to obtain far-UV CD profiles were coumpounded by the presence of high salt concentrations especailly below 210 nm. However, the signal at 222 nm appeared to be similar at different salt concentrations leading us to conclude that no significant change in secondary structure take place upon addition of NaCl.

The structural changes observed as a function of the salt concentrations in CD studies however show two different effects. At pH 7.4 the increasing salt concentration seems to induce structural rigidity around the disulfide bonds whereas at pH 2 the structure seems to get loosened around the disulfides with an increase in the salt concentration. This is typical of the Hofmeister effect where ions reverse the effect as a function of pH. In the case of Gdn.HCl studies and in the presence of low urea concentrations we observed a structure inducing effect as a function of NaCl concentration but at 7 M urea a linear loss in the structure around disulfides was observed with the addition of NaCl. Thus, we see a role reversal from pH 7.4 to 2, from hexameric form to monomeric form, or from folded form to largely unfolded form. The latter effect could also be compared to Goto et al., [67] as above.

Thus, from these comparisons it is not easy to discern the real effects taking place (neither Debye-Hückel nor Hofmeister series could be generalized) but what stands out is the relation with the structural changes and propensity to aggregate. This is further supported by the SEC and CD data. NaCl increases the dissociation of urea treated or urea untreated insulin and induces similar changes at pH 7.4 in the CD spectra. In addition, insulin populated distinct conformational states as evident in phase diagrams comparable to Gdn.HCl at lower pH. Also the observed dissociation of insulin induced with NaCl in this study is similar to dissociation induces by urea alone in our previous SEC studies [42]. From this discussion it follows that whether the effect is due to screening, specific ion-pair interaction or solvent-protein interactions, it is finally the structural change induced in the molecule that determines its propensity to fibrillate.

**Figure 5:**
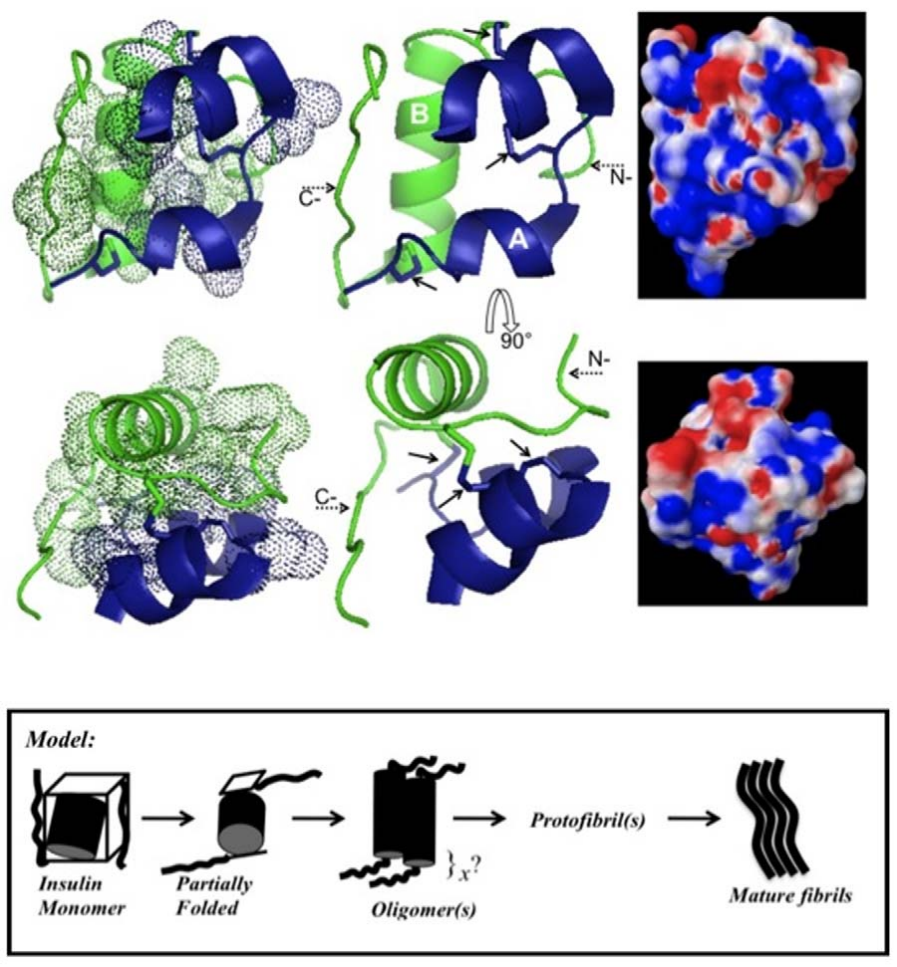
Crystal structure of insulin (PDB ID: 9INS) [**68**] with 90° rotation. Dark colored (blue) element corresponds to chain-A and light colored (green) to chain-B of insulin in the central structures. The three disulfide bond of insulin have been shown by solid arrows. The dotted arrows indicate the N- and C-terminal segments of chain-B. The dotted spheres on the left hand side of the figure show the central hydrophobic core of insulin. The surface potential of insulin molecule (blue - positive; red – negative) obtained after processing by PDB2PQR [69] and APBS [70] has been shown on the right hand side of the figure. The proposed model of insulin fibrillation is depicted at the bottom of the figure (see text for details).

From above discussion it seems plausible that the model that fits these observations could be explained by the help of insulin structure (PDB ID: 9INS) [68] shown in Figure 5. Chain-A and chain-B of insulin have been shown in dark and light color, respectively, in the center of the figure. Three disulfides of insulin shown by solid arrows lie on the surface of the molecule. On the right side are the structures exhibiting the surface potential on the insulin molecule (blue - positive; red – negative, these structures were obtained by running PDB2PQR version 1.7 [69] on 9INS followed by processing with APBS software [70]). The pictures show considerable distribution of both negative and positive charges on the surface available for interaction with counter ions. It is most eminent that these ionic interactions lead to the subtle changes in structure around the surface exposed disulfides. The induced changes can be enough to drift away the flexible elements (N- and C- terminal fragments) of insulin generating an aggregation prone state of the molecule and can further be explained by following discussion. The N- and C-terminal segment (shown with dotted arrow) of chain-B together with the charged residues in the helices enclose a highly hydrophobic core of the molecule (shown as the dotted spheres on the left side of Figure 5). It has been reported by H/D exchange studies that insulin possesses 10 buried protons belonging to this inner core [71] and when the core is exposed by subtle changes in the C-terminal segment of chain-B, it leads to immediate aggregation of insulin into fibrils [43]. This claim is supported by studies on truncated despentapeptide insulin (removed B26–B30 residues corresponding to C-terminal segment of Figure 5) that undergoes faster fibrillation than the full length insulin [72]. In addition, when the mobility of B-chain C-terminal is restricted by tethering it to N-terminal of chain-A, a slow transformation of insulin into amorphous aggregates instead of amyloid fibrils is observed [73]. N-terminal of B-Chain is unstructured and in similar spatial arrangement as C-terminal and thus may behave in a similar manner (in tandem or in a co-operative manner) as C-terminal. Studies from Prof. Weiss's group [74] have extensively detailed the detachment of N-terminal of both A- and B-chains of insulin in inducing insulin aggregation. Based on these facts and our data we suggest a model of insulin amyloid formation under these conditions as shown in Figure 5. Insulin has been shown as a three-dimensional polar cubic layer covering a hydrophobic core with the help of N- and C-terminals of B-chain in this model. Due to slight disturbances in mobile termini a modified form of insulin as a partially folded intermediate is generated. The modified structure contains most of the folded contacts but with the inner hydrophobic core exposed. Thus, the structural change triggers instantaneous hydrophobic inter-molecular association of more than one insulin molecule. The association proceeds through assembly into oligomers and protofibrils (containing unknown number of monomeric insulin units) before transforming into mature amyloid fibrils. The model compares well with the earlier studies indicating the involvement of aggregation prone hydrophobic core in insulin amyloid formation [72], [75], [76]. This model also compares well with the proposed model of insulin assembly obtained from studying insulin fibrils by cryo EM (Fig. 5 in ref [77]).

A review of published data indicates abundant instances depicting structural transformations in proteins induced by salts. Addition of monovalent salts has been shown to lead to global compaction of glucose oxidase rendering it resistant to proteolysis and denaturation [65]. In the case of dialkylglycine decarboxylase, simple exchange of monovalent salt anions actuated a structural switch involving just two surface residues that changed the enzyme activity and induced changes in the quaternary structure [78]. Alcohol dehydrogenase I has been reported to be stabilized by NaCl, CaCl_2_ and MgCl_2_ through inter-domain electrostatic interactions of the protein [79]. In another study, similar salts have been observed to increase the conformational stability of RNase T1 through interaction with surface exposed charged residues [80]. Besides direct interaction with surface exposed charges, salts can also induce structural changes through allosteric mechanism. Hexameric insulin (insulin at pH 7.4 in our studies) in itself has been found to undergo allosteric transition between three conformational states (T_6_, T_3_R_3_, and R_6_) by ligands and by the coordination of anions to the surface exposed His-10 in the B-chain [81]. Interestingly, this study also reports structural changes in N-terminal of B-chain (residues 1–8 that undergo coil-to-helix transformation) up on changes in the coordination of metal attached to surface exposed residue His-10. This finding becomes more relevant since one of the disulfides in insulin involves residue Cys-7 of N-terminal in B-chain (Figure 5). These observations greatly favor our proposed model.

Thus, in conclusion, we propose that fibrillation induced by salts like NaCl is better explained by the property of NaCl to induce subtle structural changes in the molecule that push aside the N- and C-terminal segment leading to the exposure of highly hydrophobic inner core of insulin. This small structural tweaking of insulin by NaCl, thus, recruits monomeric units of insulin for initial inter-molecular hydrophobic clumping that in turn leads to rapid and more complex association of insulin into amyloid fibrils. Keeping in mind that interaction of salts with proteins are diverse [58], [59], [60], [61], [62], [63], [64] this effect may only be applicable to proteins like insulin, α-synuclein [60] and other not yet studied proteins. The phenomenon may also be clear by revisiting other studied proteins in light of this article.

## Supporting Information

Figure S1 **Changes in lag time of ThT kinetics with increasing NaCl at pH 2 in the presence of 0.5 M urea. Conditions were similar as reported in** **Figure 1** **.**
(TIFF)

Figure S2 **Changes in molar ellipticity at 273 nm with increasing Gdn.HCl concentration in the presence of 2 M urea.** Filled circles show the data obtained after adding Gdn.HCl to urea treated samples, incubated for 30 minutes and open circles show the data obtained after adding urea to Gdn.HCl treated samples and incubated for 30 minutes both at pH 7.4.
(TIFF)

Figure S3 **Changes in molar ellipticity at 273 nm with increasing Gdn.HCl concentration in the presence of 2 M (filled circles), 4 M (open circles) or 7 M urea (inverted triangles) all at pH 7.4.**
(TIFF)
