# Supplementary figures and images for: The Mechanism of Enhanced Insulin Amyloid Fibril Formation by NaCl Is Better Explained by a Conformational Change Model
Source: PLoS One. 2011 Nov 21;6(11):e27906. doi: 10.1371/journal.pone.0027906 (PMC3221682; doi:10.1371/journal.pone.0027906)

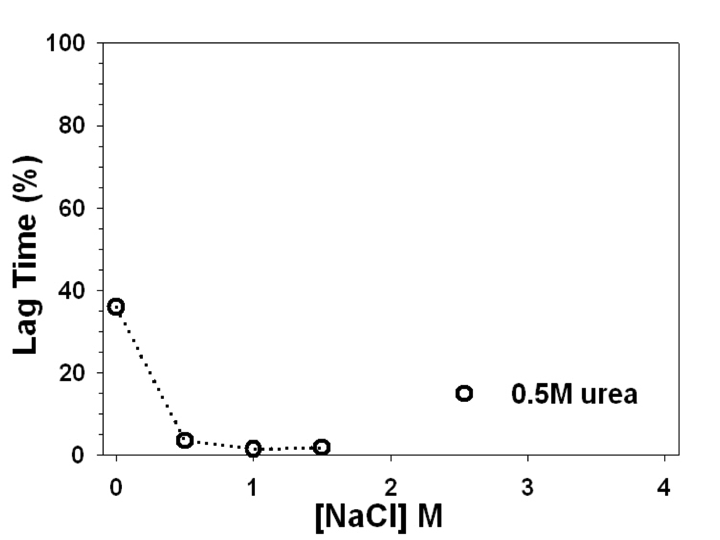

Supplement: Figure S1 — Changes in lag time of ThT kinetics with increasing NaCl at pH 2 in the presence of 0.5 M urea. Conditions were similar as reported in Figure 1 . (TIFF) [file pone.0027906.s001.tiff]

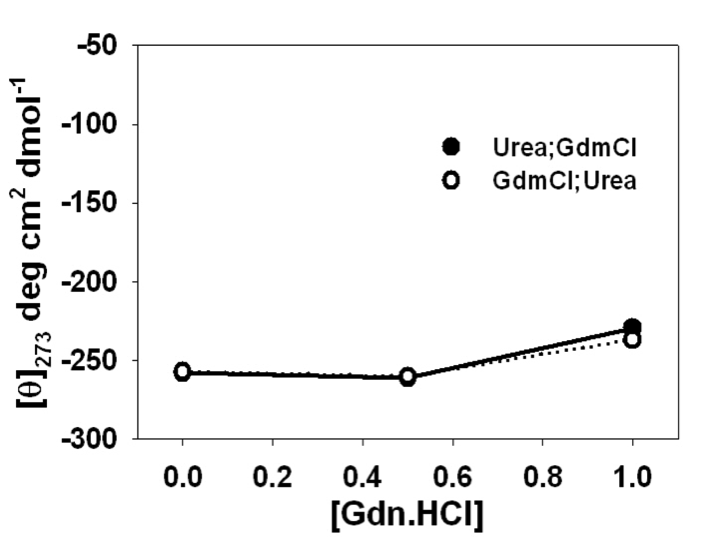

Supplement: Figure S2 — Changes in molar ellipticity at 273 nm with increasing Gdn.HCl concentration in the presence of 2 M urea. Filled circles show the data obtained after adding Gdn.HCl to urea treated samples, incubated for 30 minutes and open circles show the data obtained after adding urea to Gdn.HCl treated samples and incubated for 30 minutes both at pH 7.4. (TIFF) [file pone.0027906.s002.tiff]

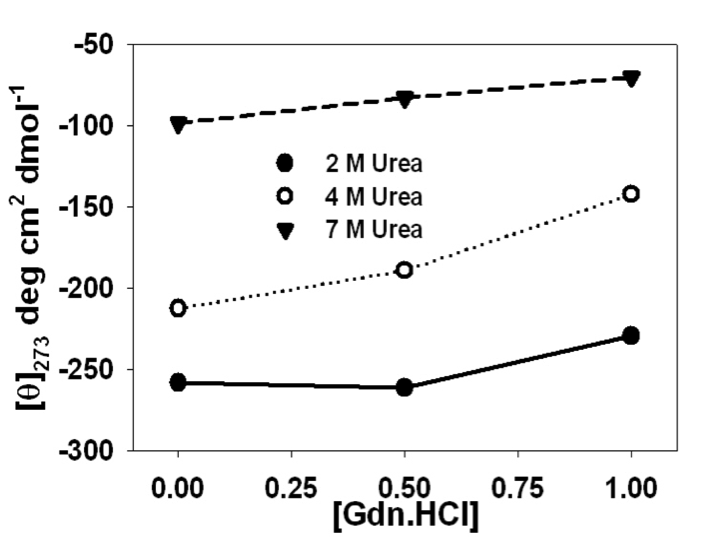

Supplement: Figure S3 — Changes in molar ellipticity at 273 nm with increasing Gdn.HCl concentration in the presence of 2 M (filled circles), 4 M (open circles) or 7 M urea (inverted triangles) all at pH 7.4. (TIFF) [file pone.0027906.s003.tiff]
